# Effects of dietary supplementation of a blend of *Saccharomyces cerevisiae*, multiple live probiotic bacteria, and their fermentation products on performance, health, and rumen bacterial community of newly weaned beef steers during a 56-d receiving period

**DOI:** 10.1093/tas/txad143

**Published:** 2023-12-21

**Authors:** Emily Treon, Taylor Sidney, Godstime Taiwo, Modoluwamu Idowu, Yarahy Leal, Deborah Ologunagba, Ibukun M Ogunade

**Affiliations:** Division of Animal and Nutritional Science, West Virginia University, Morgantown, WV 26505, USA; Division of Animal and Nutritional Science, West Virginia University, Morgantown, WV 26505, USA; Division of Animal and Nutritional Science, West Virginia University, Morgantown, WV 26505, USA; Division of Animal and Nutritional Science, West Virginia University, Morgantown, WV 26505, USA; Division of Animal and Nutritional Science, West Virginia University, Morgantown, WV 26505, USA; Division of Animal and Nutritional Science, West Virginia University, Morgantown, WV 26505, USA; Division of Animal and Nutritional Science, West Virginia University, Morgantown, WV 26505, USA

**Keywords:** beef cattle, weaning, direct-fed microbials, white blood cells

## Abstract

We examined the effects of a blend of *Saccharomyces cerevisiae*, multiple live probiotic bacteria, and their fermentation products on performance, health, and the ruminal bacterial community of newly weaned beef steers during a 56-d receiving period. Forty newly weaned Angus crossbred steers (221 ± 25.6 kg BW; 180 ± 17 d of age) were stratified by body weight (**BW**) into four pens (10 steers per pen) such that each pen had a similar average BW at the beginning of the experiment. The pens were randomly assigned to receive a corn silage basal diet (CON; *n* = 20) or the basal diet supplemented with 9 g/steer/d of PRO feed additive (PRO; *n* = 20). The PRO additive is a blend of *S. cerevisiae* and the fermentation products of *Enterococcus faecium*, *Bacillus licheniformis*, *B. subtilis*, *Lactobacillus animalis*, and *Propionibacterium freudenreichii*. The DMI and water consumed were monitored using the GrowSafe intake nodes and custom flow meters, respectively. BWs were recorded weekly to calculate average daily gain (**ADG**). Before morning feeding, 10 mL of blood was taken from each steer on days 0–7, and thereafter weekly for analyses of immune cells, plasma glucose, and NEFAs. On day 56, rumen fluid samples (200 mL each) were collected from all the steers for microbiome analysis. Over the 56-d receiving period, the supplemental PRO had no effects on DMI, water intake, or ADG. However, compared to CON, beef steers fed supplemental PRO tended to have greater ADG (*P* = 0.08) and BW (*P* = 0.07) during the first 14 d of the study. There was a treatment × day interaction (*P* ≤ 0.05) for WBC, neutrophils and monocytes over the 56 d such that beef steers fed supplemental PRO had lower blood concentrations on certain days during the first 7 d after weaning, indicating reduced inflammation or stress response. The results of the rumen microbiome analysis revealed that the relative abundance of complex fiber degrading or obligate proton-reducing bacterial genera such as *Bacteroides*, *Ruminococcus gauvreauii* group, *Desulfovibrio*, *Syntrophococcus*, and *Acetitomaculum* were greater (*P* ≤ 0.05) in beef steers fed supplemental PRO compared to CON. This study demonstrated that dietary supplementation of PRO improved the growth performance, reduced stress or inflammatory response during the initial days after weaning, and altered the ruminal bacterial community toward increased relative abundance of bacterial genera associated with improved rumen function.

## Introduction

The postweaning period is a critical stage in beef cattle production that is associated with several events including dam-calf separation, vaccinations, transportation, and pathogen exposure (Comerford, 2022). This period is accompanied by high-stress levels, primarily during the initial 7 d after separation, resulting in reduced dry matter intake (**DMI**) and compromised nutrient utilization and immune function (Pukrop et al., 2018). Several studies have shown that the health and immune function of beef steers during the first few days have significant effects on long-term productivity throughout the life of the animals (Harvey et al., 2021; Lorenz, 2021). Therefore, it is crucial to investigate the strategies that can mitigate the negative impact of weaning stress on the health and immune function of beef steers.

Numerous studies have demonstrated that dietary supplementation of direct-fed microbials (**DFMs**) such as *Saccharomyces cerevisiae* and several other bacteria including *Lactobacillus casei* and *B. subtilis* can help alleviate weaning stress and its effects on the calf during the receiving period (Phesatcha et al., 2021; Maamouri and Salem, 2022). Furthermore, DFMs are known to contain several microbial fermentation products. These serve as rich sources of nutritional metabolites that have been evaluated in several studies to improve the gastrointestinal health and performance of beef cattle (Deters et al., 2018; Hall et al., 2018; Pukrop et al., 2018). In recent years, microbial feed additives have been formulated to contain a blend of one or more microorganisms and their fermentation products to ensure efficacies and multifactorial responses (McAllister et al., 2011; Ogunade et al., 2020). In a recent study, Idowu et al. (2022) demonstrated that dietary supplementation of a multi-component microbial additive improved the immune and metabolic status of beef steers during a 35-d receiving period. However, responses to DFMs and their fermentation products are known to be inconsistent across several studies due to several factors such as strain, diet, inclusion level, and animal factors (McAllister et al., 2011). Inconsistent responses to microbial additives coupled with the ongoing advancements in microbial products highlight the necessity for additional research. Therefore, we hypothesized that dietary supplementation of a multispecies DFM would improve the DMI, health, and immune status of newly weaned beef steers. The objective of this study was to evaluate the effects of dietary blend of live *S. cerevisiae*, *Enterococcus faecium*, *Bacillus licheniformis*, *B. subtilis*, *Lactobacillus animalis*, *Propionibacterium freudenreichii*, and their fermentation products on DMI, growth, performance, health, and ruminal bacterial community of newly weaned beef steers during a 56-d receiving period.

## Materials and Methods

### Animals, Housing, and Feeding

All animal care and use procedures were in accordance with the guidelines for the use of Animals in Agriculture Teaching and Research as approved by West Virginia University (IACUC Protocol #2108046615.1). Forty newly weaned Angus crossbred steers (12-h postweaning; 221 ± 25.6 kg of body weight [**BW**]; 173 ± 11 d of age) from a single source were used. The beef cattle were vaccinated 5 mo prior to the start of the experiment and boosted 2 wk prior. The beef steers were transported ~100 miles to the research feedlot barn, and immediately weighed and processed (day 0). Processing included ear tag placement for unique animal ID, administration of appropriate vaccines, and an injection of dewormer. The vaccine protocol included Alpha-7/MB-1 Cattle Vaccine (Boehringer Ingelheim Animal Health, Duluth, GA), Pyramid-5 + Preresponse SQ Cattle Vaccine (Boehringer Ingelheim Animal Health), and the dewormer used was Safeguard Dewormer Suspension (Merck Animal Health, Summit, NJ). Based on day 0 BW, the beef steers were stratified by BW into four weight blocks. Within each weight block, the steers were randomly assigned into four pens (10 steers per pen) such that each pen had a similar average BW at the beginning of the experiment. Each pen (size = 39.0 × 32.0 ft^2^) was equipped with two GrowSafe intake nodes (GrowSafe Systems Ltd., Airdrie, AB, Canada) to measure individual feed intake. Additionally, each pen was equipped with custom flow meters (JLC International, Inc., New Britain, PA, USA) to collect water intake data. The pens were randomly assigned to receive a corn silage-based diet with no additive (CON; 2 pens; *n* = 10 per pen) or a basal diet supplemented with 9 g per head of a PRO additive (PRO; 2 pens; *n* = 10 per pen) for a period of 56 d. The PRO additive (Papillon, Easton, MD) is a blend of live *S. cerevisiae* (1.41 billion CFU/g), multiple live bacteria (*E. faecium*, *B. licheniformis*, *B. subtilis*, *L. animalis*, and *P. freudenreichii*) and their fermentation products (total bacterial count = 120 million CFU/g). The basal diet was fed as a total mixed ration (**TMR**; Table 1), and the additive was blended into the TMR at a specific percentage, calculated based on the previous day’s average feed intake for each pen (day × intake was utilized to determine the inclusion rate for day ‘x +1’). This procedure ensured that each beef steer in every pen received the required quantity of the additive, amounting to an average of 9 g (12.7 billion CFU of *S. cerevisiae* and 1.08 billion CFU of total bacteria) of PRO per head per day. To prevent any risk of cross-contamination, the CON and PRO diets were prepared separately in dedicated feed trucks. Both diets were provided ad libitum to the steers, and they had unrestricted access to water. Over the course of the experiment, about five animals experienced pinkeye or hock injuries which were promptly addressed with suitable medications administered by a licensed veterinarian.

**Table 1.** Ingredient and chemical composition of the basal diet^1^

Ingredient (% DM)	% of dietary DM
Corn silage	94.3
Concentrate supplement^2^	5.20
Vitamin and mineral premix^3^	0.50
*Nutrient analysis* ^4^	
DM, %	46.8
CP, %	13.3
aNDF, %	27.8
ADF, %	16.5
Starch, %	37.5
Ca, %	1.08
P, %	0.46
TDN, %	72.5
NE_m_, Mcal/kg	1.60
NE_g_, Mcal/kg	1.04

^1^Chemical composition of basal diet calculated from analysis and concentration of individual ingredients.

^2^Traditions 50% beef supplement (Southern States Cooperative, Richmond, VA) contained processed grain by-products, plant protein products, ground limestone, urea, salt, cane molasses, potassium sulfate, magnesium sulfate, sodium selenite, vitamin A supplement, calcium carbonate, vegetable oil, manganous oxide, vitamin D3 supplement, vitamin E supplement, zinc oxide, lecithin, phosphoric acid, basic copper chloride, magnesium chloride, propylene glycol, natural and artificial flavors, ferrous sulfate, calcium iodate, and cobalt carbonate; guaranteed analysis: 50% CP; 5% Ca; 0.55% P; 2% Na; 3.9% salt; 1% K, and 66,000 IU/kg vitamin A.

^3^Guaranteed analysis: 15% Ca; 7.5% P; 20% salt; 1% Mg; 1% K; 3,600 mg/kg Mn; 12 mg/kg Co; 1,200 mg/kg Cu; 3,600 mg/kg Zn; 27 mg/ kg Se; 60 mg/kg I; 660,000 IU/kg vitamin A; 660 IU/kg vitamin E; and 66,000 IU/kg vitamin D.

^4^Values other than DM are expressed as a percentage of dietary DM; DM, dry matter; CP, crude protein; aNDF, neutral detergent fiber (amylase treated); ADF, acid detergent fiber; NEm, net energy of maintenance; NEg, net energy of gain.

### DMI, Water Intake, and BW Measurement

The quantity of feed and water intake was monitored using the GrowSafe intake nodes and custom flow meters, respectively. A 24-h intake was measured from 0800 to 0800 hours the next day. Samples of TMR were collected weekly from both diets and were weighed and oven-dried at 55 °C for 48 h to determine dry matter content. BWs of the beef steers were obtained before morning feeding after 12 h of feed withdrawal on days 0, 14, and 56 using a regularly calibrated Tru-Test weighing scale (Cattlesoft Inc., College Station, TX) located at the working facility. Average daily gain (**ADG**) was determined by subtracting the initial weight on day 0 from the final weight on day 56 and then dividing it by the duration of the experiment (56). Additionally, the ADG for the initial 14 d and the feed-to-gain ratio for the entire 56 d were calculated.

### Blood Sample Collection

Before morning feeding (0800 hours), 10 mL of blood was taken from each steer on days 0, 1, 2, 3, 4, 5, 6, 7, and weekly thereafter. The blood samples were taken from the jugular vein into tubes containing lithium heparin and tubes containing ethylenediaminetetraacetic acid (**EDTA**; Fisher Scientific Company). The lithium heparin-containing tubes were placed on ice immediately following collection and centrifuged at 2,500 × *g* for 15 min at 4 °C for plasma preparation, then stored at −80 °C for further analysis of glucose and non-esterified fatty acids (**NEFA**). Plasma samples collected on days 0 and 56 were analyzed for NEFA and glucose concentrations in duplicate. These analyses were conducted using commercially available assays: NEFA-C kit (Wako Diagnostics Inc., Richmond, VA) for NEFA and a quantitative colorimetric kit (G7521-1L; Pointe Scientific Inc., Canton, MI) for glucose.

Immediately following each sampling (within 5 min), the whole blood samples in the EDTA tubes for all days were analyzed for white blood cells, neutrophils, lymphocytes, and monocytes using an IDEXX Procyte DX (IDEXX Laboratories Inc., Youngstown, OH).

### Rumen Fluid Collection and 16S rRNA Gene Sequencing

On day 56, rumen fluid samples (200 mL each) were collected from all the steers into polypropylene conical bottom tubes using an orally administered stomach tube connected to a vacuum pump (Ruminator, Wittibruet, Bayern, Germany). The rumen fluid samples were placed immediately on ice following collection and then stored at −80 °C and analyzed for microbiome analysis after DNA extraction. Microbial DNA was extracted from the rumen fluid samples using DNeasy PowerSoil Pro Kit (Qiagen, Catalog Number ID: 47014) following the manufacturer’s instructions. Total DNA concentration was measured using a NanoDrop 2000 spectrophotometer (Thermo Fisher Scientific, Waltham, MA). All the DNA samples had A260:A280 ratio from 1.75 to 2.1. The samples were prepared using Qiagen QIAseq phased primers designed to target the V3/V4 regions of the 16S gene, following the manufacturer’s instructions (Qiagen; catalog number: 333845). The forward primer sequence used was 5ʹ-CCTACGGGNGGCWGCAG-3ʹ, and the reverse primer sequence was 5ʹ-GACTACHVGGGTATCTAATCC-3ʹ. Subsequently, the prepared samples underwent a cleaning and normalization process before being sequenced on a v3 MiSeq 600-cycle flow cell, generating 2 × 276 bp paired-end reads.

### Data and Statistical Analysis

All growth performance data and blood parameters were analyzed as a randomized block design using the MIXED procedure of SAS (SAS 9.3, SAS Inst. Inc., Cary, NC), using beef steer as the experimental unit. The model included the fixed effects of treatment and the random effects of block (BW). Day 0 was used as a covariate, when applicable. Parameters collected repeatedly over time were analyzed using repeated measures and tested for the effect of treatment (CON vs. PRO), day of collection and the day × treatment interaction. Appropriate covariance structures were used based on the lowest Akaike values (Wang and Goonewardene, 2004). Day 0 was used as a covariate, when applicable. Results were considered significant when *P *≤ 0.05 and tendencies were declared when 0.05 > *P* ≤ 0.10.

The 16S rRNA gene sequencing data were analyzed following a previously described procedure (Idowu et al., 2023). Briefly, the initial quality control and adapter trimming of the raw sequence files were conducted using Illumina binary base call (BCL) Convert v3.9.3 with default parameters. Subsequently, the resulting fastq files were imported into Qiime2 for further analysis. Primer sequences were removed using the cutadapt plugin within Qiime2. The sequences underwent denoising via the dada2 plugin in Qiime2. The denoised sequences were then matched to operational taxonomic units (**OTUs**) employing the Silva database, with a sequence similarity threshold of 97%. This was accomplished using the VSEARCH utility within the feature-classifier plugin in Qiime2. These OTUs were consolidated into their respective taxonomic units, and their counts were converted to reflect relative frequencies within each sample. Statistical analyses of the OTU data were performed utilizing the MicrobiomeAnalyst platform (microbiomeanalyst.ca). The data were initially rarefied to the minimum library size and normalized using cumulative-sum scaling. Subsequently, this rarefied data were utilized for analyzing alpha diversity (Chao1 index) and beta diversity (Bray–Curtis distance matrix-based principal coordinates analysis or PCoA) at the genus taxonomy level. Differences in beta diversity distance were assessed using permutational multivariate analysis of variance (PERMANOVA) with 999 permutations. Differentially abundant microbial taxa between CON and PRO at the genus taxonomy level were identified using the linear discriminant analysis (**LDA**) effect size method (**LEfSe**). This method is based on a Kruskal–Wallis test with a significance level of α ≤ 0.05 and a logarithmic LDA score cutoff of 2.0 (Kruskal and Wallis, 1952).

## Results and Discussion

The actual average intake of the supplemental PRO, based on the average TMR intake (on an as-fed basis) and inclusion rate in the TMR, was 9.64 g/steer/d. Therefore, the average intake was ~107.1% of the targeted dose of 9 g/steer/d.

Table 2 presents the results of the effects of dietary supplementation of PRO on the growth performance of the beef steers. Compared to CON, beef steers fed supplemental PRO tended to have greater ADG (0.45 vs. 0.98 kg/d, *P* = 0.08) and BW (227 vs. 235 kg, *P* = 0.07) during the first 14 d of the study. However, beef steers fed CON tended to have greater (*P* = 0.07) ADG than PRO from days 15 to 56. Over the 56-d receiving period, the supplemental PRO had no effects on DMI (*P* = 0.96), water intake (*P* = 0.34), ADG (*P* = 0.66), or feed-to-gain ratio (*P* = 41). Growth performance and DMI are often compromised in the initial days following weaning due to stress factors associated with separation from the mother, transportation, dietary changes, and other elements (Arthington et al., 2013; Rauch et al., 2019). These stress factors adversely affect the animals’ immune competence and overall health, making them more susceptible to diseases (Galyean et al., 1999; Bernhard et al., 2012). Although there was no difference in DMI, the fact that supplemental PRO increased the ADG of beef steers during the initial 14 d of the study suggests potential health improvement. Previous studies have indicated that the benefits of feeding microbial additives containing *S. cerevisiae* to beef cattle are more pronounced under stress conditions, such as weaning (Duff and Galyean, 2007; Deters et al., 2018). These benefits are believed to be mediated through enhanced gastrointestinal health, which in turn supports better nutrient utilization (Ogunade et al., 2019; Oh et al., 2019). Another possible explanation for the increased ADG observed in beef steers fed supplemental PRO could be due to the positive effects of the fermentation products contained within the additive. Microbial fermentation products, which include nutritional metabolites such as organic acids, amino acids, vitamins, nucleotides, and lipids, can serve as vital nutrient sources for cattle. These nutrients can support health and enhance growth performance in stressed animals (Cruz Ramos et al., 2000). Consistent with these findings, several other studies have reported improved ADG in beef cattle during the initial days of feeding microbial additives containing either live microbes, microbial fermentation products, or both (Elam et al., 2003; Baah et al., 2009; Adeyemi et al., 2019). For instance, Baah et al. (2009) reported an increase in ADG and feed efficiency during the initial 28 d of dietary supplementary of a mixed culture of *L. casei* and *L. lactis* to beef cattle, with no significant improvements observed in the subsequent periods. In contrast, Adeyemi et al. (2019) found no significant effects during the first 21 d but noted more pronounced growth efficiency (ADG and feed efficiency) in the final 21 d of their study of beef steers supplemented with a *S. cerevisiae* blend, leading to an overall improvement in ADG. Interestingly, in both of these studies, DMI was similar, a finding consistent with the results of this current study. The variation in responses observed across different studies is possibly because DFM products are known to be inconsistent and heterogeneous across studies, with variations resulting from a range of factors including diet composition and differences in doses and microbial strains and species.

**Table 2.** Growth performance and dry matter intake of beef steers fed diet supplemented with a blend of *Saccharomyces cerevisiae* and multiple live probiotic bacteria during a 56-d receiving period

	CON	PRO	SEM	*P* value
Initial weight, kg	221	221	8.43	0.98
Body weight, kg, day 14	227^y^	235^x^	3.92	0.07
Final body weight, kg, day 56	305	302	7.54	0.66
DMI, kg/d, days 1 to 14	4.62	4.35	0.33	0.41
DMI, kg/d, days 15 to 56	7.66	7.57	0.41	0.81
DMI, kg/d, days 1 to 56	6.92	6.94	0.38	0.96
ADG, kg/d, days 1 to 14	0.45^y^	0.98^x^	0.29	0.08
ADG, kg/d, days 15 to 56	1.85^y^	1.60^x^	0.13	0.07
ADG, kg/d, days 1 to 56	1.50	1.44	0.13	0.66
Feed-to gain ratio, days 1 to 56	6.22	4.95	1.54	0.41
Water intake, L/d, days 1 to 14	11.5	10.8	0.92	0.44
Water intake, L/d, days 15 to 56	17.48	16.64	1.49	0.57
Water intake, L/d, days 1 to 56	16.3	15.1	1.26	0.34

CON, control; PRO, a blend of *Saccharomyces cerevisiae, Enterococcus faecium*, *Bacillus licheniformis*, *Bacillus subtilis*, *Lactobacillus animalis*, *Propionibacterium freudenreichii,* and their fermentation products fed at 9 g/steer/d (Papillon, Easton, MD); SEM, standard error of mean; ADG, average daily gain; DMI, dry matter intake.

^x,y^Within a row, treatment means with different superscripts tend to differ, 0.05 > *P* ≤ 0.10.

^a,b^Within a row, treatment means with different superscripts differ, *P* ≤ 0.05.

**Figure 1.**
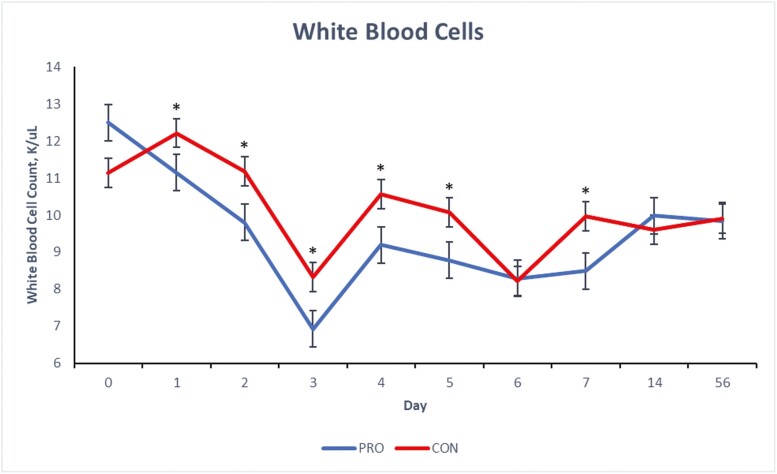
Effects of dietary supplementation of a blend of *Saccharomyces cerevisiae,* multiple live probiotic bacteria and their fermentation products on white blood cell count (K/µL) in beef steers during a 56-d receiving period. CON, control; PRO, a blend of *Saccharomyces cerevisiae, Enterococcus faecium*, *Bacillus licheniformis*, *Bacillus subtilis*, *Lactobacillus animalis*, *Propionibacterium freudenreichii,* and their fermentation products fed at 9 g/steer/d (Papillon, Easton, MD); values from day 0 were used as independent covariate for each day. *Within days: *P ≤ 0.05; SEM = 0.27; treatment × day interaction: *P* = 0.01.

**Figure 2.**
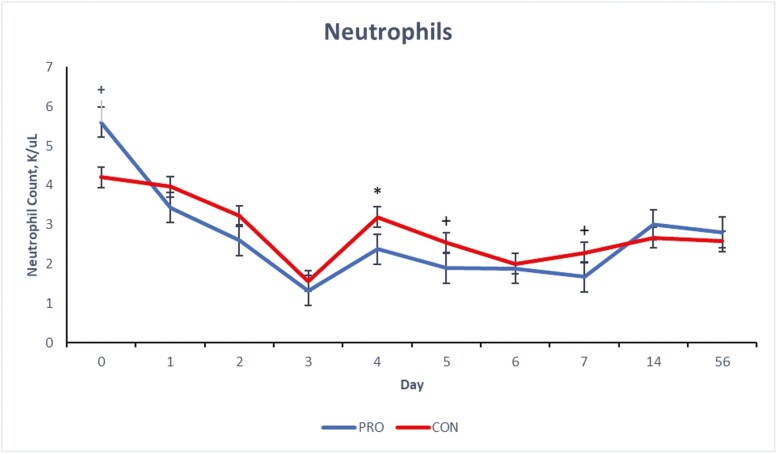
Effects of dietary supplementation of a blend of *Saccharomyces cerevisiae*, multiple live probiotic bacteria and their fermentation products on neutrophil count (K/µL) in beef steers during a 56-d receiving period. CON, control; PRO, a blend of *Saccharomyces cerevisiae, Enterococcus faecium*, *Bacillus licheniformis*, *Bacillus subtilis*, *Lactobacillus animalis*, *Propionibacterium freudenreichii,* and their fermentation products fed at 9 g/steer/d (Papillon, Easton, MD); Values from day 0 were used as independent covariate for each day. *Within days: *P ≤ 0.05, ^+^0.05 > *P* ≤ 0.10; SEM = 0.16; treatment × day interaction: *P* = 0.03.

**Figure 3.**
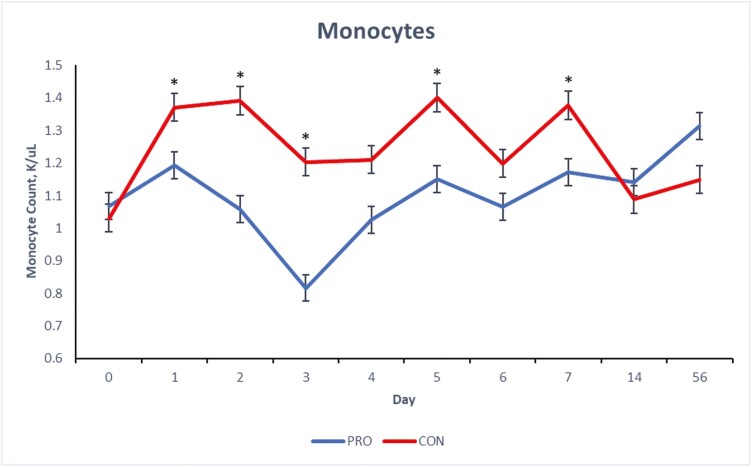
Effects of dietary supplementation of a blend of *Saccharomyces cerevisiae*, multiple live probiotic bacteria and their fermentation products on monocyte count (K/µL) in beef steers during a 56-d receiving period. CON, control; PRO, a blend of *Saccharomyces cerevisiae, Enterococcus faecium*, *Bacillus licheniformis*, *Bacillus subtilis*, *Lactobacillus animalis*, *Propionibacterium freudenreichii,* and their fermentation products fed at 9 g/steer/d (Papillon, Easton, MD); values from day 0 were used as independent covariate for each day. *Within days: *P ≤ 0.05, ^+^0.05 > *P* ≤ 0.10; SEM = 0.04; treatment × day interaction: *P* = 0.0002.

**Figure 4.**
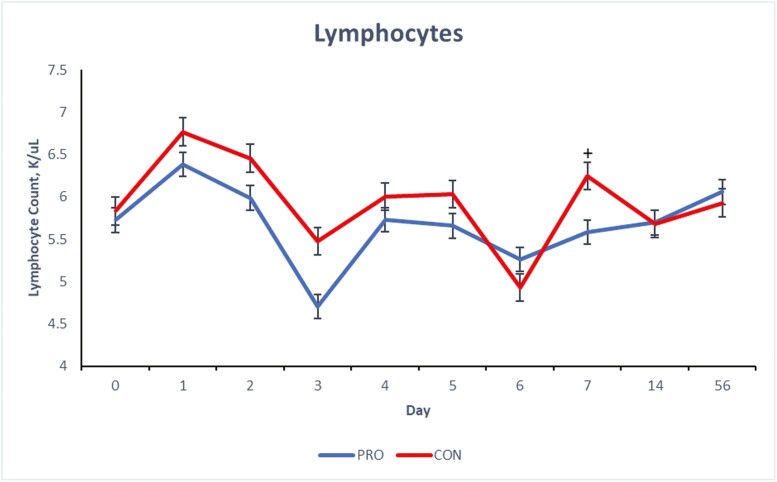
Effects of dietary supplementation of a blend of *Saccharomyces cerevisiae*, multiple live probiotic bacteria and their fermentation products on lymphocyte count (K/µL) in beef steers during a 56-d receiving period. CON, control; PRO, a blend of *Saccharomyces cerevisiae, Enterococcus faecium*, *Bacillus licheniformis*, *Bacillus subtilis*, *Lactobacillus animalis*, *Propionibacterium freudenreichii,* and their fermentation products fed at 9 g/steer/d (Papillon, Easton, MD); values from day 0 were used as independent covariate for each day. *Within days: *P ≤ 0.05, ^+^0.05 > *P* ≤ 0.10; SEM = 0.16; treatment × day interaction: *P* = 0.08.

There were treatment × day interactions (*P* ≤ 0.05) for blood concentrations of total WBC, neutrophils and monocytes of the beef steers over the 56-d period (Figures 1, 2, and 3) and a tendency (*P* = 0.09) for lymphocyte concentration (Figure 4). Compared to CON, beef steers fed supplemental PRO had lower (*P* ≤ 0.05) blood concentrations of monocytes and total WBC during the first 7 d after weaning (with exceptions on days 4 and 6; *P* > 0.05, for monocyte and WBC, respectively). However, these effects were not observed on days 14 and 56. Neutrophil count was or tended to be lower (*P* ≤ 0.10) in the beef steers fed the PRO diet on days 4, 5, and 7 compared to the CON group, though the counts were similar on other days. Lymphocyte count was similar for all days except on day 7 when it was lower (*P* = 0.09) in beef steers fed supplemental PRO.

White blood cells, such as neutrophils, lymphocytes, and monocytes, are fundamental to the immune system. Elevated counts of these cells can indicate inflammation, stress, or infection responses. While the observed concentrations of WBC, neutrophils, lymphocytes, and monocytes for both groups fall within the standard reference range for healthy beef cattle (Roland et al., 2014), a value at the higher end of this range might suggest subtle inflammatory stress. The stress induced by weaning and other associated changes like diet and housing can disrupt the animals’ physiological balance, especially in the days immediately following weaning (Blecha et al., 1984; Hulbert and Moisá, 2016). White blood cell count is a commonly used marker for detecting inflammatory responses (O’Loughlin et al., 2011). Previous research has indicated that neutrophilia often emerges as a primary biomarker of inflammatory stress in beef cattle postweaning (Lynch et al., 2012; O’Loughlin et al., 2012). The reduced blood concentration of WBCs, monocytes, and neutrophils in the beef steers fed supplemental PRO during the initial postweaning period suggests a potentially reduced inflammatory stress response in these animals. The components of the supplemental PRO (*S. cerevisiae*, live bacteria, and their fermentation products such as glucans, mannan, and other nutritional factors) are known to improve gut health and immunity due to their immunomodulatory effects (Taiwo et al., 2020; Sun et al., 2021) which might play a role in modulating the immune response during stress events like weaning.

No effects of dietary PRO supplementation were observed for the plasma glucose and NEFA concentrations of the beef steers at the end of this study (*P* > 0.05; Table 3). Plasma glucose and NEFA concentrations are frequently used to evaluate the energy balance of ruminants, which is primarily influenced by DMI. The absence of effects may be attributed to similar DMI and/or the diet provided in this study, which met the energy requirements of the beef steers. Another possible explanation for the lack of effects is that the plasma glucose and NEFA concentrations were only analyzed on the last day of the experiment when the weaning stress levels of the beef steers had diminished, as indicated by similar WBC and neutrophil counts. Consistent with our results, in a 30-d feeding trial on beef cattle supplemented with *S. cerevisiae*, Zhang et al. (2022) found no alterations of NEFAs in serum. In another study, Sun et al. (2021) observed no differences in NEFA, β-hydroxybutyrate, glucose, or triglycerides for treatment; however, they did find a time interaction for glucose. Similarly, Olagaray et al. (2019) reported no differences between *S. cerevisiae* fermentation product supplemented cattle and control cattle in plasma concentrations of free fatty acids, β-hydroxybutyrate, glucose, or insulin.

**Table 3.** Glucose and NEFA concentrations of beef steers fed a diet supplemented with a blend of *Saccharomyces cerevisiae* and multiple live probiotic bacteria during a 56-d receiving period

	CON	PRO	SEM	*P* value
Glucose, mg/dL	79.4	84.5	3.21	0.12
NEFA, µM	190	172	24.0	0.47

CON, control; PRO, a blend of *Saccharomyces cerevisiae, Enterococcus faecium*, *Bacillus licheniformis*, *Bacillus subtilis*, *Lactobacillus animalis*, *Propionibacterium freudenreichii,* and their fermentation products fed at 9 g/steer/d (Papillon, Easton, MD); SEM, standard error of mean.

**Figure 5.**
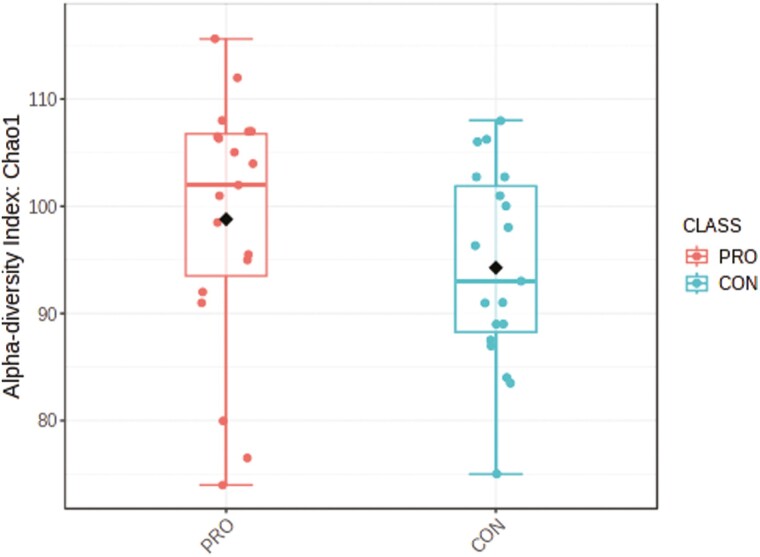
Alpha diversity index (Chao1 index; *P* value = 0.19) of the rumen bacterial community of beef steers fed diet supplemented with a blend of *Saccharomyces cerevisiae*, multiple live probiotic bacteria and their fermentation products. CON, control; PRO, a blend of *Saccharomyces cerevisiae, Enterococcus faecium*, *Bacillus licheniformis*, *Bacillus subtilis*, *Lactobacillus animalis*, *Propionibacterium freudenreichii,* and their fermentation products fed at 9 g/steer/d (Papillon, Easton, MD).

**Figure 6.**
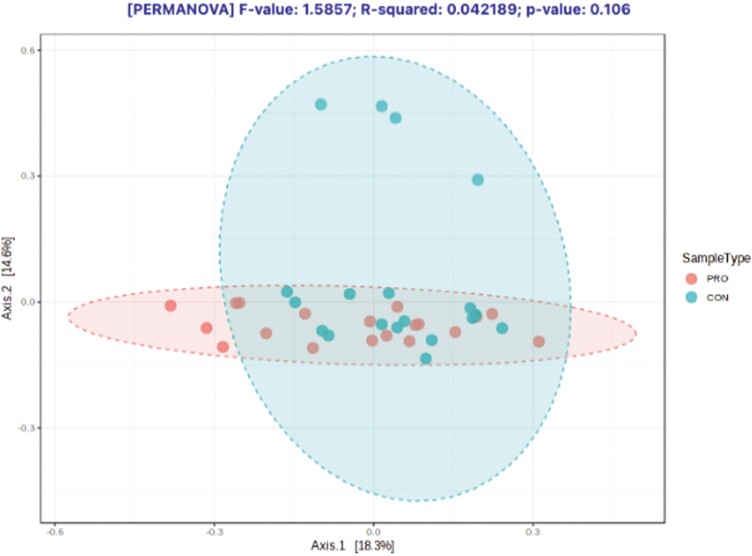
Beta diversity (Bray–Curtis-PCoA based on unweighted Unifrac distance) of the rumen bacterial community of beef steers fed diet supplemented with a blend of *Saccharomyces cerevisiae*, multiple live probiotic bacteria and their fermentation products (PERMANOVA; *P* = 0.11). CON, control; PRO, a blend of *Saccharomyces cerevisiae, Enterococcus faecium*, *Bacillus licheniformis*, *Bacillus subtilis*, *Lactobacillus animalis*, *Propionibacterium freudenreichii,* and their fermentation products fed at 9 g/steer/d (Papillon, Easton, MD).

**Figure 7.**
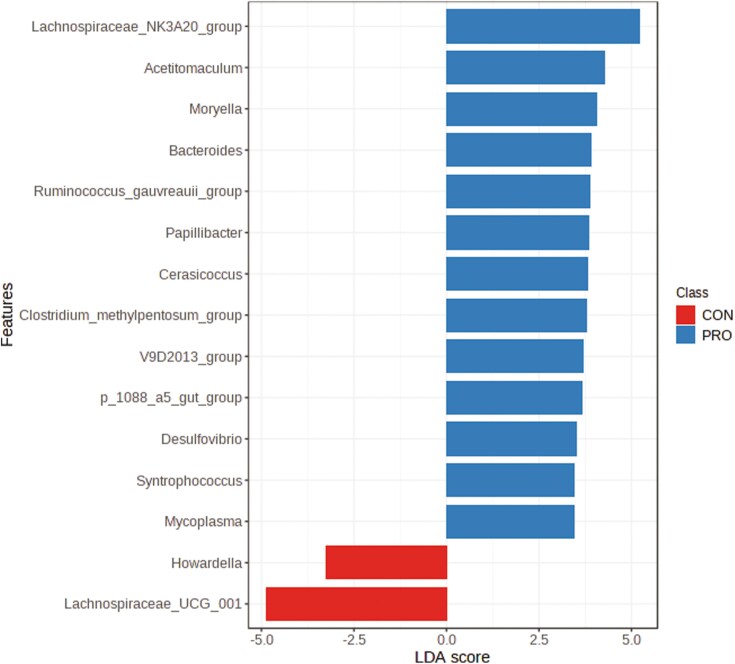
Differentially abundant bacterial taxa at the genus level determined using LDA effect size analysis in beef steers fed diet supplemented with a blend of *Saccharomyces cerevisiae*, multiple live probiotic bacteria and their fermentation products. CON, control; PRO, a blend of *Saccharomyces cerevisiae, Enterococcus faecium*, *Bacillus licheniformis*, *Bacillus subtilis*, *Lactobacillus animalis*, *Propionibacterium freudenreichii,* and their fermentation products fed at 9 g/steer/d (Papillon, Easton, MD).

There was an average of 250,873 ± 46,912 read pairs per sample. The rarefaction curves showed that the rate of increase in OTU number slowed down with increasing reads per sample and tended to plateau, illustrating that the sequencing coverage was adequate (Supplementary Figure S1). The Chao1 index (a measure of alpha diversity) was similar (Figure 5; *P* = 0.19) for both groups, as was the PLS-DA score plot (Figure 6; *P* = 0.11) based on an unweighted Unifrac distance (a measure of beta diversity). The LEfSe results (Figure 7) showed that the relative abundance of *Lachnospiraceae NK3A20* group, *Acetitomaculum*, *Moryella*, *Bacteroides*, *Ruminococcus gauvreauii* group, *Papillibacter*, *Cerasicoccus*, *Clostridium methylpentosum* group, V9D2013 group, p-1088-a5 gut group, *Desulfovibrio*, *Syntrophococcus*, and *Mycoplasma* was greater (LDA ≥ 2.0; *P* ≤ 0.05) in beef steers fed supplemental PRO compared to CON, whereas the relative abundance of *Howardella* and *Lachnospiraceae* UCG 001 was lower (LDA ≥ 2.0; *P* ≤ 0.05).

The modes of action of DFMs vary depending on several factors, including the type of DFM; however, most DFMs are known to modify the ruminal environment by altering the ruminal microbial composition (Ban and Guan, 2021). Bacteria belonging to the *Lachnospiraceae NK3A20* group and *Lachnospiraceae* UCG 001 are known to produce butyrate, which can help stimulate the functional development of the ruminal epithelium (Chun et al., 2021; Yin et al., 2021). For unknown reasons, the relative abundance of *Lachnospiraceae* NK3A20 group was increased while that of *Lachnospiraceae* UCG 001 was reduced by PRO supplementation. *Desulfovibrio* and *Syntrophococcus* are obligate proton-reducing acetogens that can compete against methanogens for H_2_, thereby reducing ruminal methane emissions as a by-product of fermentation (Friedman et al., 2017; Levy and Jami, 2018; Zhao and Zhao, 2022). Although methane emission was not analyzed in this study, an in vitro study demonstrated that the extract of *S. cerevisiae* increased hydrogen utilization by acetogens and decreased methane production (Chaucheyras et al., 1995). In a similar study, Lila et al. (2004) reported that a strain of *S. cerevisiae* decreased methane emission after a 24-h incubation in a batch culture system. However, multiple in vivo studies, including a meta-analysis, demonstrated that *S. cerevisiae* and fermentation products of *Bacillus* and *Aspergillus* did not reduce CH_4_ production in dairy and beef cattle (Chung et al., 2011; Darabighane et al., 2019; Oh et al., 2019).

*Papillibacter* and *R. gauvreauii* group are Gram-positive bacteria in the family *Ruminococcaceae*, a family of bacteria known to degrade complex plant materials to volatile fatty acids in the rumen. *Bacteroides* are one of the most abundant fiber-degrading anaerobes in the rumen (Flint and Duncan, 2014; Chen et al., 2022). Bacteria belonging to *Acetitomaculum* and *Moryella* can ferment formate, glucose, cellobiose, fructose, and other carbohydrates produced from fiber degradation to volatile fatty acids, which are important energy sources for ruminants (Gualdrón-Duarte and Allen, 2018; Yu et al., 2020). The increased relative abundance of the aforementioned bacterial genera in beef steers fed a diet supplemented with PRO may indicate an enhanced ruminal health and function.

## Conclusion

The results of this study demonstrated that feeding supplemental PRO to newly weaned beef steers did not affect the DMI, ADG, and feed efficiency of the beef steers over the 56-d receiving period. Nevertheless, the inclusion of the feed additive led to a decrease in the overall concentration of total WBC, neutrophils, monocytes, and lymphocytes in whole blood during the initial 14 d following weaning. This reduction indicates a potential mitigated stress and inflammatory response during this critical postweaning period. In addition, dietary supplementation of PRO increased the relative abundance of some bacterial genera involved in fiber degradation and improved rumen development. Overall, this study provides valuable insights into the potential benefits of PRO supplementation for newly weaned beef steers, particularly during the initial days after weaning. Further research is necessary to explore the underlying mechanisms and optimize the use of this additive under various beef cattle management conditions.

## Supplementary Material

txad143_suppl_Supplementary_Figures_S1
