# Supplementary material for: Effects of dietary supplementation of a blend of Saccharomyces cerevisiae, multiple live probiotic bacteria, and their fermentation products on performance, health, and rumen bacterial community of newly weaned beef steers during a 56-d receiving period
Source: Transl Anim Sci. 2023 Dec 21;8:txad143. doi: 10.1093/tas/txad143 (PMC10785767; doi:10.1093/tas/txad143)
Supplement: txad143_suppl_Supplementary_Figures_S1 [file txad143_suppl_supplementary_figures_s1.docx]

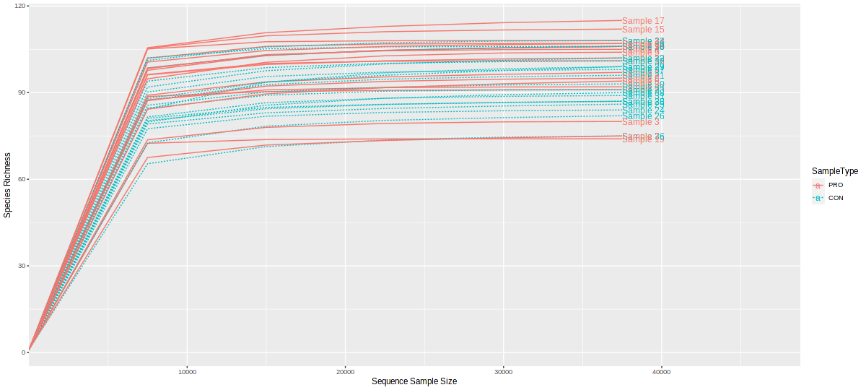


Supplemental Figure 1. Rarefaction curve of all the samples

CON = control; PRO = a blend of *Saccharomyces cerevisiae, Enterococcus faecium*, *Bacillus licheniformis*, *Bacillus subtilis*, *Lactobacillus animalis*, *Propionibacterium freudenreichii* and their fermentation products fed at 9 g/steer/d (Papillon, Easton, MD).
